# Editorial: Stem cells and extracellular vesicles in bone health, disease and regeneration, volume II

**DOI:** 10.3389/fendo.2025.1739689

**Published:** 2025-12-22

**Authors:** Chao Ma, Jianfei Liang, Shengfeng Bai, Liming Zhao, Bingdong Sui

**Affiliations:** 1 State Key Laboratory of Oral & Maxillofacial Reconstruction and Regeneration & National Clinical Research Center for Oral Diseases & Shaanxi International Joint Research Center for Oral Diseases, Center for Tissue Engineering, School of Stomatology, The Fourth Military Medical University, Xi’an, Shaanxi, China; 2 Key Laboratory of Shaanxi Province for Craniofacial Precision Medicine Research, College of Stomatology, Xi’an Jiaotong University, Xi’an, China; 3 Department of Implant Dentistry, College of Stomatology, Xi’an Jiaotong University, Xi’an, China; 4 Institute for Stem Cell Biology and Regenerative Medicine, Stanford University School of Medicine, Stanford, CA, United States; 5 Department of Orthopaedic Surgery, Tongji Hospital, Tongji Medical College, Huazhong University of Science and Technology, Wuhan, China

**Keywords:** bone, extracellular vesicles, mesenchymal stem cell, osteoporosis, regeneration

Bone diseases and bone defects significantly affect human health, imposing a considerable medical burden on society (1–4). However, currently available therapeutic strategies for these conditions are neither cost-effective nor easily accessible (5). Notably, medications used to treat osteoporosis are associated with rare complications such as osteonecrosis of the jaw and atypical femoral fracture (depending it on long term therapy), while effective methods for bone regeneration fall short in addressing severe infections and large-scale defects (6). In preclinical studies, various sources of stem cells, particularly mesenchymal stem cells (MSCs), have been utilized in the treatment of osteoporosis and the promotion of bone regeneration, yielding certain positive outcomes (1). Furthermore, extracellular vesicles (EVs) have garnered extensive attention recently for their potential in treating bone diseases due to their cell-free nature, low immunogenicity, and capacity to carry a diverse array of effective molecules and drugs (1, 7). This Research Topic aims to explore and summarize the most cutting-edge advancements in this field. It seeks to provide the latest foundational insights into bone diseases while contributing to efforts aimed at maintaining optimal bone health.

## Bone disease and regeneration

With the progression of aging, osteoporosis, a prevalent manifestation of skeletal senescence closely associated with impaired bone homeostasis, inevitably occurs. MSCs as the most critical type of stem cells in bone tissue, undergo senescence, a key factor contributing to the development of osteoporosis. The article by Tong et al. elaborates on both the causes and mechanisms underlying MSC senescence while also summarizing cutting-edge theories and technologies aimed at inhibiting or eliminating senescent cells to mitigate osteoporosis. Notably, this article emphasizes innovative techniques utilizing stem cells and EVs for the treatment of osteoporosis, highlighting their promising therapeutic efficacy. Therefore, a deeper understanding of the mechanisms governing MSC senescence will enhance our comprehension of the physiological and pathological characteristics associated with osteoporosis, thereby facilitating the development of more targeted and specific therapeutic strategies.

Previous preclinical studies have demonstrated the therapeutic potential of MSCs in osteoporosis; however, their clinical application is hindered by safety and ethical concerns. In contrast, MSC-derived EVs, as a cell-free therapeutic strategy, have garnered increasing attention in the scientific community due to their favorable safety profile and low immunogenicity (8). In their study, Zhang et al. conducted the first systematic review and meta-analysis to evaluate the preclinical efficacy of MSC-EVs in osteoporosis. Their findings indicate that MSC-EVs consistently exert beneficial effects across multiple preclinical models, offering robust evidence for their translational potential. Specifically, MSC-EV treatment was associated with significant improvements in bone mineral density, bone mass, microarchitectural parameters, bone remodeling markers and biomechanical properties in osteoporotic animal models. These results underscore the importance of further research to expand and refine the existing evidence base, thereby supporting the therapeutic promise of MSC-EVs in the management of osteoporosis.

The health, disease, and regeneration of bone are not governed solely by intrinsic skeletal factors, but rather unfold within a dynamic network of crosstalk and reciprocal interactions with multiple organs and tissues throughout the body. Metabolic disorders can disrupt the equilibrium between bone formation and resorption, leading to conditions such as osteoporosis, and further impair bone defect repair through chronic inflammation and lipotoxicity. A comprehensive understanding of the molecular mechanisms by which metabolic networks regulate bone cell function and mediate inter-organ communication will establish a systemic framework for treating bone-related diseases and enhancing bone regeneration, thereby supporting the development and clinical translation of metabolism-targeted therapeutic strategies. In addition to MSC-derived EVs, extracellular vesicles from diverse tissue sources have demonstrated therapeutic potential in osteoporosis, as illustrated by brown adipose tissue-derived EVs (BEVs) reported by Zhang et al. This study is distinguished by its innovative conceptual framework: rather than focusing exclusively on local skeletal factors, it investigates the inter-organ metabolic crosstalk between the liver and bone. The authors first establish a mechanistic link between dysregulated lipid metabolism and jaw osteoporosis. Subsequently, they demonstrate that administration of BEVs effectively ameliorates lipid metabolic abnormalities and, importantly, concurrently alleviates jaw osteoporosis—thereby achieving a dual therapeutic benefit. By elucidating the role of systemic metabolic dysregulation in bone pathology, this work advances our understanding of the comorbidity between metabolic disorders and skeletal diseases. Furthermore, it proposes a novel therapeutic strategy, leveraging BEVs to simultaneously target metabolic dysfunction and osteoporosis, offering a paradigm shift toward systemic, multi-target interventions.

Beyond degenerative disorders such as osteoporosis, post-injury bone regeneration impacts a significantly broader population. Tendon-bone injuries, for instance, predominantly affect younger individuals, yet effective therapeutic options remain limited. To address this unmet clinical need, Jiang et al.’s study focuses on the regulation of inflammation during the tendon-bone healing process. It posits that inflammation exerts a dual role in tissue regeneration: while acute inflammatory responses facilitate necrotic tissue clearance, tenocyte proliferation, and collagen fiber deposition, prolonged or excessive inflammation can exacerbate tissue damage and promote fibrotic scarring. Therefore, spatiotemporal control of the inflammatory response is critical for optimal tendon-bone interface repair. Notably, the article highlights MSCs as potent modulators of immune activity, capable of enhancing regenerative outcomes. Furthermore, EVs are emphasized for their anti-inflammatory and pro-angiogenic properties, which may synergistically support tissue remodeling and integration at the injury site. Consequently, EV-based cell-free therapies represent promising, safer, and potentially more effective strategies for tendon-bone repair, meriting rigorous preclinical and clinical evaluation.

## Current limitations

However, several methodological bottlenecks currently hinder translational applications in this field. In MSC research, the host’s pathological or injured microenvironment significantly compromises therapeutic efficacy, impairing the sustained survival and functional integration of transplanted cells. Immunogenicity remains a concern in allogeneic settings, and prolonged *in vitro* expansion may lead to spontaneous malignant transformation of MSCs. For EV research, although extracellular vesicles are generally considered to exhibit lower immunogenicity than whole MSCs, xenogeneic EVs may carry MHC molecules or residual components from culture media, potentially triggering immune rejection. Moreover, the absence of standardized protocols for EV sources—such as those derived from different MSC subtypes—and isolation methods leads to substantial batch-to-batch variability in composition. EVs also suffer from limited long-term storage stability, and the key molecular mechanisms underlying their role in bone regeneration remain incompletely understood. Furthermore, translational development of MSC- and EV-based therapies faces regulatory and ethical challenges. Global regulatory frameworks for these therapies are inconsistent, with no universally established approval pathways for gene-edited cell products. Ethical concerns regarding cell sourcing and genetic manipulation persist. Insufficient long-term safety data, combined with incomplete reporting of critical outcomes in certain clinical trials, further undermines reproducibility. These limitations not only constrain scientific credibility but also impede the clinical translation of promising findings.

## Future perspective

Encouragingly, recent advances in gene editing technologies, artificial intelligence (AI), and personalized precision medicine have created new opportunities for MSC and EV applications in treating bone-related disorders. For example, CRISPR-Cas9 enables precise modulation of osteogenic transcription factors—such as RUNX2 and SP7—and immunoregulatory genes in MSC or EV-producing cells, enhancing osteogenic potential while minimizing risks associated with insertional mutagenesis. AI models trained on multi-omics datasets—including transcriptomic and proteomic profiles—can predict the performance of MSCs or EVs under varying scaffold compositions, bioactive molecule loadings, and microenvironmental conditions, enabling rapid design of personalized regenerative formulations and reducing reliance on empirical optimization. Additionally, well-designed multi-arm, multicenter clinical trials that concurrently evaluate diverse delivery routes, cell sources, and phenotypic modulation strategies can enhance data robustness and accelerate regulatory validation.

In summary, the studies featured in this Research Topic explore the mechanisms of osteoporosis driven by MSC senescence and propose corresponding intervention strategies. They also examine the therapeutic potential of MSC-EVs and BEVs in osteoporosis, emphasizing how a systemic understanding of bone biology can yield novel mechanistic insights and advance translational approaches. Furthermore, MSCs have also been explored for the regeneration of tendon-bone interface injury. Collectively, these contributions synthesize recent cutting-edge progress in the use of MSCs and EVs for degenerative bone diseases and bone defects, elucidating their modes of action and highlighting their clinical promise in managing skeletal disorders.
